# THE HEALTHY AGING INDEX AND ITS ASSOCIATION WITH MORTALITY IN OLDER MEXICAN AND EUROPEAN AMERICANS

**DOI:** 10.1093/geroni/igz038.3481

**Published:** 2019-11-08

**Authors:** Vinutha Ganapathy, Mini E Jacob, Meghan I Short, Mitzi M Gonzales, Claudia Satizabal Barrera, Sudha Seshadri, Chen-pin Wang, Helen P Hazuda

**Affiliations:** 1 Glenn Biggs Institute for Alzheimer's and Neurodegenerative Diseases, U T Health San Antonio, San Antonio, Texas, United States; 2 UT Health San Antonio, San Antonio, United States; 3 University of Texas Health Science Center, Texas, United States; 4 U T Health San Antonio, San Antonio, Texas, United States

## Abstract

Mexican Americans (MA) have higher morbidity compared to European Americans (EA); however, the mortality rate remains lower (Hispanic Paradox). The Healthy Aging Index (HAI) captures clinical and subclinical morbidity in older adults and is useful in examining ethnic differences in mortality for a given disease burden. We assessed the association between baseline HAI and all-cause mortality over 12 years of follow-up among older MAs (n=394) and EAs (n=355) in the San Antonio Longitudinal Study of Aging (SALSA) and examined differences between ethnic groups. HAI incorporates non-invasive measures (systolic blood pressure, forced vital capacity, creatinine, fasting plasma glucose (FPG), Mini-Mental State Exam). Missing baseline data for HAI components and covariates were imputed using multiple imputations. Proteinuria was used instead of creatinine due to non-availability. Scores of 0, 1, 2 were given from lowest to highest tertile HAI categories; diagnosis of diabetes, hypertension, and renal failure were included in the highest tertiles. Cox proportional hazards models estimated the association between HAI and mortality, adjusting for confounders. After adjusting for age, gender, education, income, BMI, smoking and ethnicity, HAI was independently associated with mortality (HR 1.25 (1.16-1.35), p-value <0.0001). We found no interaction effect between HAI and ethnicity on mortality ((p-value for interaction = 0.78). In the SALSA sample, HAI is a predictor of mortality after adjusting for confounders in both MAs and EAs. The absence of a significant HAI*ethnicity interaction effect further demonstrated that HAI works equally well as a predictor of mortality in both MAs and EAs.

